# Case Report: Treatment and management of a child at high risk of caries

**DOI:** 10.3389/fped.2023.1103386

**Published:** 2023-10-23

**Authors:** Wen Fan, Qianjing Chen, Yushan Zhang, Jixian Feng, Yuankun Zhai, Baojie He

**Affiliations:** ^1^ School of Stomatology, Henan University, Kaifeng, China; ^2^ Henan University Seth Stomatological Hospital, Zhengzhou, China; ^3^ Department of Stomatology, Weinan Central Hospital, Weinan, China; ^4^ Department of Stomatology, the Second Affiliated Hospital of Shandong First Medical University, Tai'an, China; ^5^ Kaifeng Key Laboratory of Periodontal Tissue Engineering, Kaifeng, China

**Keywords:** caries risk assessment, caries management, high caries risk, premature eruption, case report

## Abstract

**Introduction:**

Caries are at the forefront of childhood diseases. Although childhood caries is usually not life-threatening, it can affect children's dental–maxillofacial development and mental health and place significant financial and psychological burdens on parents. As the focus of childhood dental caries shifts to early diagnosis and prevention rather than restorative dentistry alone, screening children at a high risk of dental caries is urgent. Appropriate caries prevention measures and treatment sequences can effectively reduce the occurrence and development of dental caries in children.

**Case:**

We report the case of a 7-year-old boy presenting with a high risk of dental caries involving multiple primary teeth and premature eruption of the permanent teeth. We shifted the caries status of the child from high to moderate likelihood. At the 9-month post-treatment follow-up, the patient had no new dental caries, and the length and width of the dental arch were effectively maintained.

**Conclusion:**

Oral health education, dental plaque removal in a regular basis, and fluoride application contribute to caries management.

## 1. Introduction

Caries is a disease caused by ecological changes in the oral biofilm flora. Frequent intake of carbohydrates stimulates the symbiotic homeostatic microbiome to become dominated by abiogenic, aciduric bacteria (1). Changes in biofilm activity lead to an imbalance between demineralization and remineralization, resulting in the demineralization of hard tissue in the tooth, the primary clinical manifestation of caries (2). Childhood caries, a common oral disease in children, is characterized by a wider range of lesions, more rapid progression, and harm than in adults, leading to adverse effects on children's oral health, dental–maxillofacial development, and general health. Many factors influence oral health in children, including diet, oral hygiene, tooth position and morphological characteristics, salivary composition and flow rate, fluoride, economic factors, nutrition, genetic factors, and gene–environment interactions (3). The dental caries prevalence rate among children in China is rising; thus, oral health needs improvement, according to the Fourth National Health Epidemiological Survey 2018 released by the National Health Commission (4). With the updated caries etiology, the concepts of caries treatment have changed. Clinically, caries treatment varies according to the degree of caries risk, activity, and depth. When caries is detected early (i.e., initial stages of demineralization are usually manifested as white spot lesions in the enamel), it can be controlled using preventive measures, such as diet counseling, oral hygiene instructions, and fluoride application. Fluoride is utilized in various forms, including water fluoridation, toothpaste, foam, gel, varnish, and other applications. Caries risk assessment (CRA) and the controlling factors are a substantial part of pediatric caries management. CRA refers to assessing the possibility of caries occurrence and changes in the degree of caries within a certain period (5). CRA requires an evaluation of caries sensitivity to develop an individualized oral healthcare plan by developing detailed conditions, dietary habits, oral hygiene habits, and oral health status. The significance of CRA is that if an oral healthcare provider can detect caries at the earliest stage, caries-related defects can be prevented. In 2002, the American Academy of Pediatric Dentistry (AAPD) developed the CRA tool (CAT). Children aged >6 years old are considered at high risk for caries if they meet one of the criteria in Table 1 (6). We report a case of detailed treatment of a child with a high risk of caries, aiming to propose a reasonable treatment sequence and appropriate treatment measures to achieve a better therapeutic effect**.** The child had severe dental caries, where each deciduous tooth was decayed, filled, or missing because of caries. We successfully prevented the child from developing additional caries through caries management.

**Table 1:** Caries-risk assessment form for ≥6 years old. (For dental providers)

Factors	High risk	Moderate risk	Low risk
Biological			
Patient is of low socioeconomic status	Yes		
Patient has >3 between-meal sugar-containing snacks or beverages per day	Yes		
Patient has special healthcare needs		Yes	
Patient is a recent immigrant		Yes	
Protective			
Patient receives optimally fluoridated drinking water			Yes
Patient brushes teeth daily with fluoridated toothpaste			Yes
Patient receives topical fluoride from health professional			Yes
Additional home measures (e.g., xylitol, MI paste, and antimicrobial)			Yes
Patient has dental home/regular dental care			Yes
Clinical findings			
Patient has ≥1 interproximal lesions	Yes		
Patient has active white spot lesions or enamel defects	Yes		
Patient has low salivary flow	Yes		
Patient has defective restorations		Yes	
Patient wearing an intraoral appliance		Yes	

Circling those conditions that apply to a specific patient helps the practitioner and patient/parent understand the factors that contribute to or protect from caries. Risk assessment categorization of low, moderate, or high is based on the preponderance of factors for the individual. However, clinical judgment may justify using one factor (e.g., ≥1 interproximal lesion and low salivary flow) in determining overall risk.

Overall assessment of the dental caries risk: High □ Moderate □ Low □.

## 2. Case description

A 7-year-old Chinese boy reported to our clinic with the chief complaint of mandibular posterior dental caries, which had persisted for 3 years. One month prior, he had undergone temporary restoration for cavitation in mandibular posterior teeth, and his mobile deciduous teeth 51, 61, 54, and 64 were extracted 1 week before. The child had a mixed dentition, with sealed pits and fissures on teeth 16, 26, 36, and 46. The buccal groove of tooth 36 was filled. The CAT for children ≥6 years old was used for CRA at the first visit. The child was classified as a high-caries-risk type because of the intake of refined sugar snacks and drinks between meals (at least thrice daily), biological factors, and interproximal caries lesions ≥1. He brushed his teeth once daily for less than 2 min without flossing. He was breast- and bottle-fed until the age of 3 years and had a habit of drinking milk at night. These oral behaviors suggested poor control of the plaque biofilm. Teeth 83, 84, and 85 had undergone root canal therapy (RCT) because of apical infectious foci. The boy's parents denied having systemic diseases or a history of drug allergies. The boy was overweight, of average height, and attended primary school. His parents were civil servants of medium socioeconomic status and lived in a third-tier city with uncrowded housing conditions.

**Figure 1:**
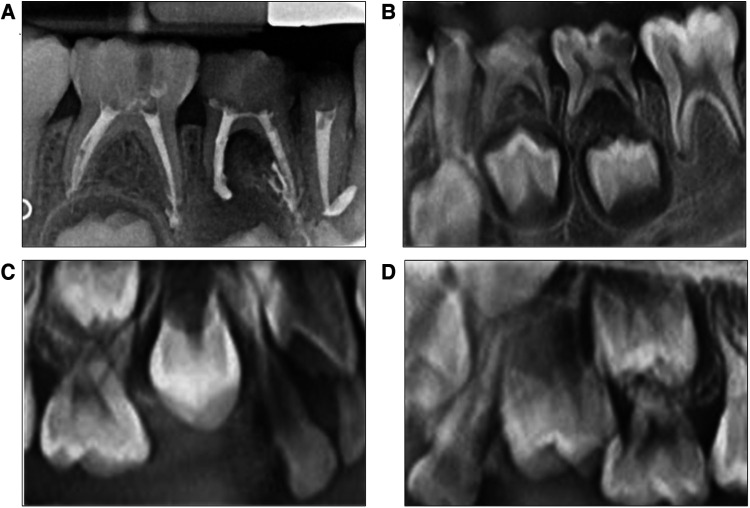
(**A**–**D**) are X-rays before treatment. (**A**) is the bitewing radiograph of teeth 83, 84, and 85. Periapical inflammation of tooth 84 is extended to the lower permanent tooth embryo. (**B**) is the bitewing radiograph with teeth 73, 74, and 75 presenting severe caries. Periapical inflammation of tooth 75 extended to the lower permanent tooth embryo. (**C**,**D**) are x-ray images that show no root image in teeth 14 and 24.

A physical intraoral examination showed early caries lesions as scattered yellowish-brown spots on the tooth surfaces. The radiographs shown in Figure 1 were obtained during this visit. The mesio–occlusal–distal surfaces of teeth 84 and occlusal and buccal surfaces of teeth 85 were filled, without abnormalities in the mucous membranes in the apical areas. Tooth 83 had a filled root stump, without any mobility or abnormality in the apical areas.

### Radiographic findings

Tooth 85 showed a root canal resistance projection image up to the root tip, whereas the apical root filling of the mesial root canal had overflowed, without periapical transmission image. Tooth 84 had a root canal internal resistance image up to the root tip, with a distal periapical transmission image affecting the hard bone plates of permanent tooth germ 44, accompanied by a mesial apical root filling overflow image. Tooth 83 had a dense root canal resistance image, an apical root filling overflow, and no apical transmission (Figure 1A). The tooth surfaces of 73^M, D^, 74^MOD^, and 75^DO^ showed blackened cavities. These cavities were firm when probed, eliciting no pain, evoking no discomfort on cold stimulation, and exhibiting no percussion pain. Teeth 73, 74, and 75 had no abnormalities in the apical mucosae. On the x-ray image, the crown of tooth 73 showed a transmission image reaching the middle layer of dentin without periapical abnormalities. The crown of tooth 74 was in the transmission image near the pulp cavity with a visible low-density image in the root furcation. In contrast, the permanent tooth germ of tooth 34 maintained hard bone plate integrity without periapical abnormalities. The crown of tooth 75 showed a transmission image near the pulp cavity, with a visible low-density image in the root furcation. A low-density image was observed around the distal root apex, affecting the hard bone plate of the permanent tooth germ of tooth 35 (Figure 1B). Teeth 14 and 24 showed the crown but not the root images (Figures 1C,D). Teeth 53^D^, 55^O, P^, and 65^O, P^ had caries that were firm on probing without eliciting pain and exhibited no discomfort on cold stimulation or percussion pain. The radiographic finding revealed a transmission image on the crown of tooth 53, reaching the middle layer of the dentin without periapical abnormalities. Teeth 52 and 62 showed grade Ⅱ mobility, with yellowish-brown palatal surfaces, and were hard on probing. Tooth 63 also had brownish-yellow patches on the surface and was hard on probing. The gingiva was pink and firm to the touch. Sticky debris was attached to his labial and lingual cervical one-third of the teeth in both arches. The bilateral first molars were in a distal step occlusion. His decayed, missing, and filled teeth (dmft) index was 16.

Thus, the boy was diagnosed as a high-caries-risk patient. Teeth 83 and 85 were diagnosed with dental defects (after RCT); tooth 84 with chronic apical periodontitis (after RCT); teeth 74 and 75 with chronic apical periodontitis; tooth 53 with deep dental caries; teeth 55, 65, and 73 with medium caries; teeth 52, 62, and 63 with superficial caries; and teeth 14 and 24 with premature eruption.

Given the complex situation, discussions with informed consent were conducted in great detail, encompassing the treatment plans for the parents of the child and outlining the costs and potential risks. We developed a treatment strategy to reduce the risk of caries based on the following stages of illness: prevention, control, functional recovery and reconstruction, and follow-up, using the therapeutic concept of general oral practice.

### 2.1. Disease prevention stage

The child and his parents received oral health education and instructions on brushing, flossing, and fluoridation. Diet control was implemented by decreasing the frequency of snacks and the consumption of fermentable carbohydrates. During the follow-up appointments, a plaque-disclosing agent was used to detect the status of full-mouth plaque control, showing plaques in the whole dentition. The gingiva was red and slightly swollen, and bleeding was observed on probing. All tooth surfaces were polished, and oral health education was given again.

### 2.2. Disease control stage

RCT was performed on teeth 74 and 75, and resin composite restorations were placed on teeth 53, 55, 65, and 73. At the request of the parents, our patient agreed that the upper premolar should be allowed to erupt unimpededly. During the follow-up, teeth 14 and 24 were modified into a light-biting state to correct the premature contact with the opposing teeth.

### 2.3. Functional recovery and reconstruction stage

The crowns of teeth 74, 75, 83, 84, and 85 were restored.

### 2.4. Follow-up maintenance stage

Root development was observed in teeth 14 and 24, and any required occlusion adjustments were performed, followed by a 3-month follow-up to evaluate the efficacy of the treatment. Plaque control was the top priority during subsequent visits. We applied fluoride (3M™ Clinpro™ White Varnish) every 3 months, although the drinking water of the boy was already fluoridated (the fluoride concentration is 1.0 mg/L in tap water in China) (7). Timely fissure sealing of the permanent teeth with deep pits and grooves was performed on teeth 14 and 24 during treatment.

### 2.5. Follow-up and outcomes

**Figure 2:**
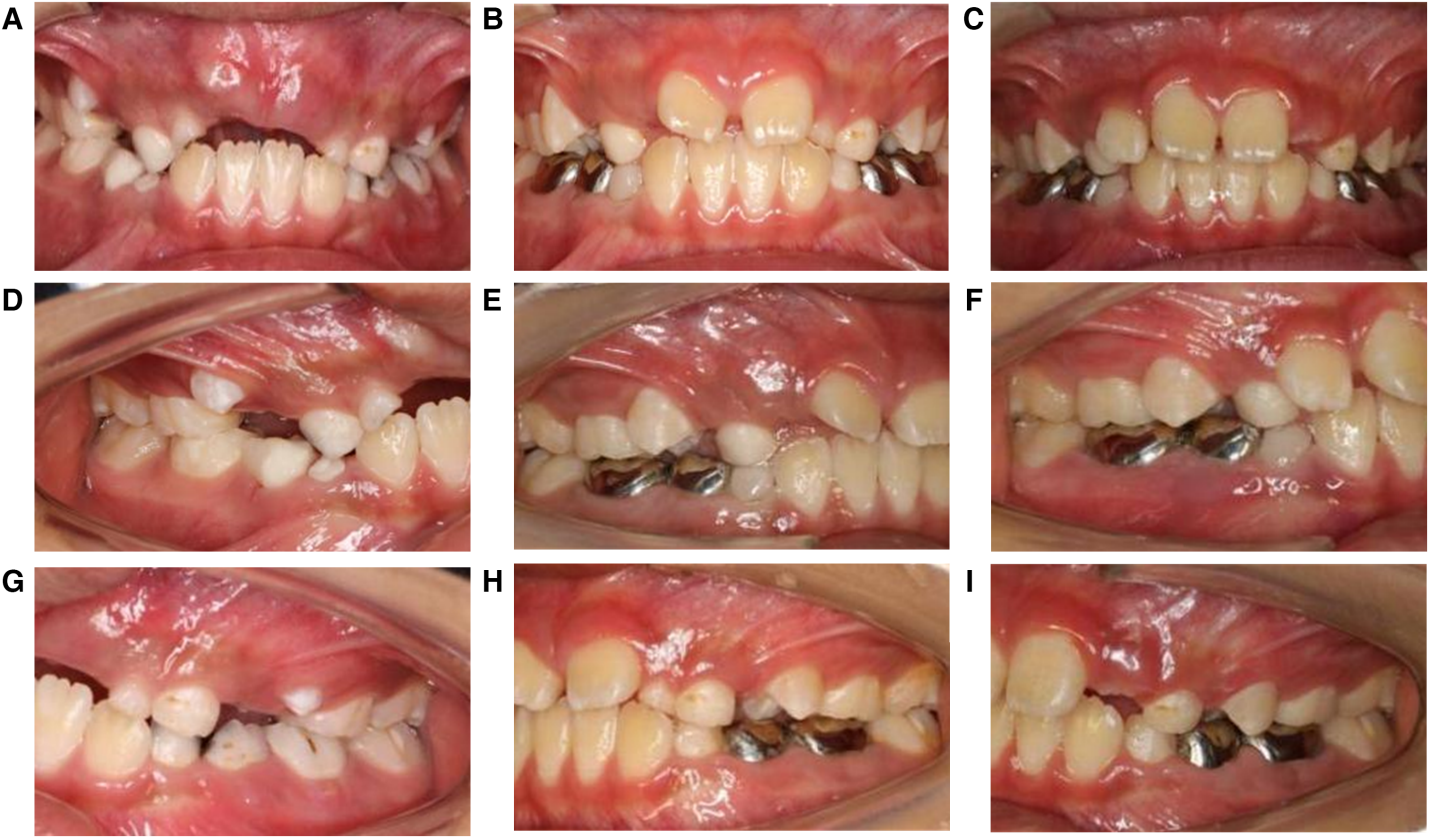
(**A**–**I**) are images of teeth before and after treatment. (**A**–**C**) are frontal view photos before and 4 months and 9 months after treatment. Teeth 11 and 21 are under eruption. (**D**–**F**) are the occlusal photos of the right side before and 4 months and 9 months after treatment. Tooth 14 is under eruption and has good contact with its opposing teeth during the follow-up. (**G**–**I**) are the occlusal photos of the left side before and 4 months and 9 months after treatment. Tooth 24 is under eruption and has good contact with its opposing teeth during follow-up.

**Figure 3:**
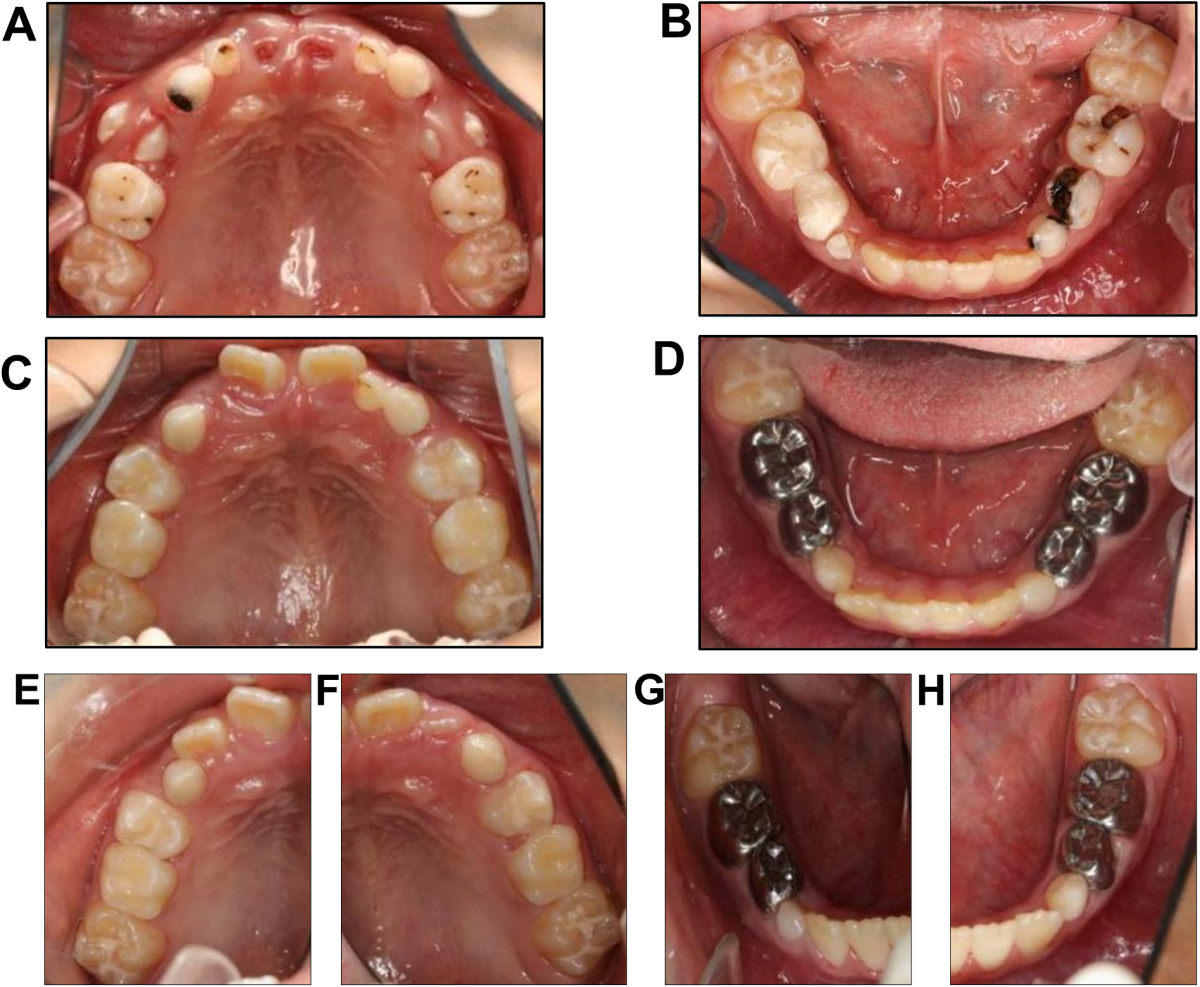
Photos of the maxillary and mandibular dentitions before treatment. (**A**,**B**) show maxillary and mandibular dentitions before treatment. (**C**,**D**) show maxillary and mandibular dentitions 4 months after treatment. (**E**–**H**) show maxillary and mandibular dentitions 9 months after treatment.

**Figure 4:**
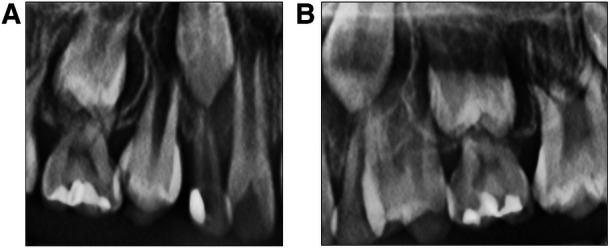
(**A**) and (**B**) are X-rays of teeth 14 and 24 after treatment. (**A**) is the X-ray of tooth 14 after 9 months of treatment. (**B**) is the X-ray of tooth 24 after 9 months of treatment.

After treatment and follow-up, teeth 12, 11, 21, and 22 erupted without caries. Intra-arch alignment and functioning of the crowns of teeth 74, 75, 83, 84, and 85 were adequate. No new dental caries were seen in the oral cavity. The lengths and widths of the dental arches were maintained. The follow-up period lasted 9 months. The restorations of teeth 53, 55, and 65 were intact without color changes. The marginal fitness and strength of the crowns of teeth 73, 74, 75, 84, and 85 were satisfactory. The preformed crown of tooth 74 was twisted, and the gum was swollen along with the distal gingiva of tooth 24 (Figure 2). During follow-up, tooth 14 was in the process of erupting and had good contact with its opposing teeth from the right occlusal view, and tooth 24 was undergoing eruption and had moderate contact with its opposing teeth from the left occlusal view (Figure 3). We hypothesized that in mixed dentition, changes in occlusion induced by the eruption of tooth 24 caused tooth twist of tooth 74, resulting in plaque accumulation and swelling of the gingival papillae. The length of root development in teeth 14 and 24 was approximately one-third of the root length at 9 months after treatment (Figure 4). Oral health education was provided to the parents and child at each visit. Changes were made to the dental healthcare practices of the boy and the awareness of oral healthcare of the parents. For instance, with his parents’ prompting, he increased his daily tooth brushing from once to twice a day, brushed more carefully, and reduced the quantity of snacks and beverages consumed between meals. Unfortunately, we subsequently lost contact with the boy and his parents.

### 2.6. Conclusion

A 9-month follow-up period is insufficient for assessing the success of caries prevention and intervention since the boy's teeth were still growing and developing. Long-term supervision is needed over the years. The treatment prioritizes improving the patients’ oral health education and oral hygiene above halting disease progression, functional recovery, and reconstruction. We report a case with a short follow-up period to emphasize the importance of caries prevention and management during mixed dentition. Children at this age begin to attend school with a certain amount of self-control and responsible behavior but still require the supervision of parents and teachers. Oral diseases are preventable or treatable with minimal intervention if diagnosed and treated early. Personalized management of dental caries should be implemented based on the life cycle of managing dental caries for different risk factors and levels. To establish a treatment plan, dental professionals must identify and evaluate the risk of caries based on a thorough comprehension of its etiology and pathology. Simultaneously, caries activity must be evaluated with a unique plan that addresses prevention, non-operative treatment, and repair therapy to restore the balance of oral microecology and management of the illness course. To standardize decision-making and treatment approaches, dental professionals should include risk assessment and management of dental caries in regular oral health services.

## 3. Discussion

In the present report, deciduous teeth were either decayed or filled because of caries. We prevented further worsening of these lesions and reduced the occurrence of caries. We conducted the diagnosis and treatment according to a reasonable treatment sequence and correct treatment measures to achieve a better treatment effect. The treatment course prioritized improving the patient’s oral health knowledge and hygiene over controlling the disease progression, functional recovery, and reconstruction. We changed the patient's oral health awareness and behavior, as he switched from brushing casually to brushing carefully and reducing the consumption of snacks and beverages. The boy's parents’ oral health literacy changed. The good oral hygiene of the boy served as evidence that his parents possessed sufficient knowledge of proper brushing and flossing techniques, enabling them to instruct him effectively. Unfortunately, we lost contact with them after he had no new dental cavities, as they did not value routine dental checkups.

According to a 2015 study, China had the second-highest direct cost of oral disorders worldwide (8). Most people in China visit the dentist only when they experience oral issues like toothaches (usually because of advanced dental caries) (9). We speculate that it is because of a general lack of awareness of oral health knowledge, dental fear, expensive dental care, and a lack of time because of busy schedules. Dentists should provide children with preventive treatments such as fissure sealants, which are more beneficial and significantly less expensive than invasive treatments (10).

Enhancing oral health knowledge among children and their patients is particularly crucial. The current philosophy of caries management is more conservative and preventive, focusing on detecting non-cavitated lesions early, identifying individual risks of caries progression, understanding the disease process, actively supervising, applying preventive measures, and carefully monitoring the disease status (11). In addition to the involvement of dentists, patients’ attention and adjustments in behaviors are also necessary for disease prevention. Concrete and clear instructions on brushing and flossing are provided for plaque removal in the clinic. Parents can detect plaque in their children using a testing fluid purchased online to check the effect of brushing at home. In China, dental fillings remain the primary treatment for tooth decay (12). However, this cannot change the etiology or correct the risk factors, resulting in a likelihood of caries re-occurrence. Previous caries experience shows that reducing caries activity is the key to prevention (13). Promoting oral health while reducing the formation of microbial membranes on tooth surfaces can be achieved by reducing the risk factors for tooth decay and increasing the protective factors.

A personalized management plan for dental caries that considers the entire management life cycle of dental caries and the different risk factors and levels of management of the periods of pre-pregnancy, pregnancy, neonatal, infant, preschool, school, adolescence, middle age, menopause, old age, and terminal life should be implemented. During pre-pregnancy and pregnancy, a minority of expectant mothers obtain oral health information from gynecologists/obstetricians/nurses in China (14). Prenatal education of parents can be the start of early preventative methods for childhood caries, and adequate dental care and oral hygiene practices during pregnancy can lessen or postpone early childhood caries (15). Children should be properly fed during the neonatal and infant periods (<3 years old) until their deciduous teeth erupt. Their teeth should be brushed every morning and evening with a suitable age-appropriate toothbrush dipped in clean water. Depending on the dentist's assessment of the child's oral health and caries risk at the first oral examination, a fluoride-containing toothpaste can be used in a “smear” amount under supervision (6). During the preschool period, children should consume a high-fiber diet to enhance chewing function. They should avoid eating before bed after brushing their teeth, undergo oral health checkups every 6 months, use fluoride locally to prevent deciduous tooth caries, and learn to brush their teeth under parental supervision using a pea-sized amount of toothpaste to ensure its effectiveness (16). During the school years (>6 years old), children should use 0.5% fluoride toothpaste twice daily. Those at a high risk of developing dental caries should undergo radiography every 6 months, undergo follow-up appointments every 3 months, and have professional local fluoride applications every 3 months to prevent cavities and repair all dental caries, as recommended by AAPD (17). Caries Management by Risk Assessment (CAMBRA) proposed antibacterial therapy (using a chlorhexidine rinse once a day for 1 week every month) and fluoride therapy (twice-daily high-concentration fluoride toothpaste and xylitol gum or mints four times daily) for patients aged 6 years through adult (18). The regimen proposed by CAMBRA has more specific recommendations than that of AAPD. During the gradual eruption of permanent teeth, the fossa and groove sealing of permanent molars and premolars was completed successively. Topical fluoridation can effectively reverse the progression of enamel plaque in deciduous and permanent teeth (19). Fluoride can help the remineralization process of hydroxyapatite and is most abundant in the outer layer of enamel (20). However, it should be noted that local long-term application of high concentrations of fluoride can lead to the growth of fluorine-resistant strains of *Streptococcus mutans* (21).

Guiding the awareness and understanding of personal responsibility is imperative for the long-term oral health of children. Dental professionals are aware that deficiencies in compliance can affect outcomes across all treatment decisions. As an approach that motivates patients to make positive behavioral changes, motivational interviewing is gaining popularity among patient-centered clinicians (13). More health education efforts are needed to contend with unhealthy oral habits, such as frequent intake of sugary food without flossing. It involves persuasive, supportive, and argumentative components and is designed for the intrinsic motivation strategies of patients (22). New media technologies need to be used, such as WeChat (a chat application in China) and databases, to conduct corresponding behavior management, health education, dentist–patient interaction, and communication to maximize control of new dental caries in daily life (23).

We shifted the child's caries status from high to moderate likelihood, according to the International Caries Classification and Management System (ICCMS™) (Table 2), and no new dental caries were found in the mouth during the 9-month follow-up (24). A limitation of this report was the short follow-up period, indicating that oral health is not well understood among patients in China. It calls for an alteration from conventional restorative to preventive dentistry.

**Table 2:** ICCMS™ caries risk likelihood matrix.

Patient risk status	Current caries status at the patient level		
Based upon the judgment of a dentist or a member of the dental team (including history of restorations and extractions and review of key risk factors)			
	No active caries lesions	Initial-stage active caries lesions	Moderate or extensive active caries lesions
Low risk	Low likelihood	Moderate likelihood	Moderate likelihood
Moderate risk	Low likelihood	Moderate likelihood	High likelihood
High risk	Moderate likelihood	High likelihood	High likelihood

## 4. Patient perspective

Dental caries is preventable through oral health education, self-care, and simple, evidence-informed, cost-effective universal measures. Oral health is vital in maintaining oral function and essential for diet and speech development (25).

## Data Availability

The original contributions presented in the study are included in the article/Supplementary Material, further inquiries can be directed to the corresponding authors.
